# 4473 Immune control of plasma cell disorders – in-depth analysis of Sox2 immunity in MGUS

**DOI:** 10.1017/cts.2020.402

**Published:** 2020-07-29

**Authors:** Sandra Patricia Susanibar Adaniya, Casey L. Cummins, Miren L. Baroja, Beatriz Carreno, Gerald P. Linette, Alfred L. Garfall, Maya G. Robnett

**Affiliations:** 1 University of Pennsylvania School of Medicine

## Abstract

OBJECTIVES/GOALS: We aim to identify and characterize anti-Sox2-specific CD8+ T cell responses in stable MUGS patients expressing HLA class I alleles-A*02:01 and /or -B*07:02. METHODS/STUDY POPULATION: Cross sectional study of patients with stable MGUS defined as stable serum paraprotein for ≥ 12 months from the MM Research Clinic at the Abramson Cancer Institute. Sox2 T cell reactivity will be assessed by IFN-γ ELISPOT assays. Rested PBMC will be pulsed with candidate Sox2-derived peptides predicted to display high affinity to HLA class I alleles and known to be processed and presented as determined by “targeted MS/MS” (mass spectrometry). The presence of anti-Sox2-specific CD8+ T cells will be confirmed in peptide/HLA multimer assays using flow cytometry. Anti-Sox2-specific CD8+ T cells will be characterized for HLA restriction and TCR αβ composition. RESULTS/ANTICIPATED RESULTS: Our work is still in progress. From Aug to Dec 2019, 22 MGUS subjects have been analyzed, 11 of which were found to have the HLA of interest. Positive Sox-2 reactivity by ELISpot was found in 3 subjects. DISCUSSION/SIGNIFICANCE OF IMPACT: Anti-Sox2 immune responses may maintain MGUS in a clinical indolent state by eliminating Sox2-expressing clonogenic MM cells. A detailed characterization of anti-Sox2 T cells followed by in-vivo assessment of their anti-myeloma activity could provide the foundation for a Sox2 based immunotherapy approach in MM.

